# Assessment of the Effect of Interferon-Beta1a Therapy on Thyroid and Salivary Gland Functions in Patients with Multiple Sclerosis Using Quantitative Salivary Gland Scintigraphy

**DOI:** 10.4274/mirt.53825

**Published:** 2014-06-05

**Authors:** Seval Erhamamcı, Bahriye Horasanlı, Ayşe Aktaş

**Affiliations:** 1 Başkent University Faculty of Medicine, Department of Nuclear Medicine, Ankara, Turkey; 2 Başkent University Faculty of Medicine, Department of Neurology, Ankara, Turkey

**Keywords:** Multiple sclerosis, thyroid diseases, salivary gland diseases, scintigraphy, interferon-beta1

## Abstract

**Objective:** Interferon-beta (IFN-β) is widely used in patients with multiple sclerosis (MS), a demyelinating disease of the central nervous system. High incidence of thyroid dysfunction has been reported after administration of IFN-β in MS patients. The aim of this study was to assess the effect of IFN-β1a therapy on simultaneous thyroid and salivary gland function in patients with MS using quantitative salivary gland scintigraphy (QSGS).

**Methods:** Fifteen relapsing-remitting (RR) MS patients treated with IFN-β1a and two control groups consisting of 15 untreated RRMS patients and 20 healthy age and sex-matched individuals were included in the study. The functional status of the salivary and thyroid glands was analysed with the QSGS and laboratory tests, including thyroid function and thyroid antibody. After intravenous administration of 150 MBq Tc-99m pertechnetate, dynamic study was performed for 25 minutes. Salivary gland secretion was stimulated with oral lemon juice at 15 minutes. At the end of dynamic study, a static image in the same projection was taken. Uptake ratios at 12-14 min (UR%) and stimulated excretion fraction (EF%) of each parotid and submandibular gland were calculated automatically from SGS. Thyroid uptake ratio (TUR) of thyroid gland was calculated from the static image.

**Results:** All MS patients treated and untreated with IFN-β1a, and healthy individuals were euthyroid. Anti-thyroid peroxidase antibody (anti-TPO) was detected in 4 out of 15 MS patients (26.6%) treated with IFN-β1a. There was no significant differences in the UR, EF and TUR values among MS patients treated and untreated with IFN-β1a, and healthy controls (p>0.05). Although the TUR values in MS patients treated with IFN-β1a were less than those of the both control group, the difference was not statistically significant (p>0.05).

**Conclusion:** IFN-β1a therapy was demonstrated to have no effect on thyroid and salivary gland functions using QSGS in patients with MS. Thyroid and salivary gland functions were also found to remain unchanged in untreated MS patients.

## Abstract (tr)

**Amaç:** Santral sinir sisteminin demiyelinizan bir hastalığı olan multipl skleroz (MS) hastalarında interferon-beta (IFN-β) yaygın olarak kullanılır. MS hastalarında IFN-β verilmesinden sonra yüksek oranda tiroid fonksiyon bozukluğu bildirilmiştir. Bu çalışmanın amacı, MS hastalarında IFN-β1a tedavisinin tiroid ve tükrük bezi fonksiyonları üzerindeki etkisinin eşzamanlı olarak kantitatif tükrük bezi sintigrafisi (KTBS) ile değerlendirilmesidir.

**Yöntem:** IFN-β1a ile tedavi edilen 15 relapsing-remitting (RR) MS hastası ve tedavi edilmemiş 15 RRMS hastası ile yaş, cinsiyet uyumlu 20 sağlıklı bireyden oluşan iki kontrol grubu çalışmaya dahil edildi. Tükrük ve tiroid bezlerinin fonksiyonu, KTBS ve tiroid fonksiyon ile tiroid antikorlarını içeren laboratuvar testleriyle analiz edildi. Yüz elli MBq Tc-99m perteknetatın intravenöz verilmesinden sonra, dinamik çalışma 25 dakika süreyle gerçekleştirildi. Tükrük bezi sekresyonu, 15. dakikada da oral limon suyu ile uyarıldı. Dinamik çalışmanın sonunda, aynı projeksiyonda statik bir görüntü alındı. Her bir parotis ve submandibuler bezin 12-14 dk tutulum oranları (%UR) ve uyarılmış ekskresyon fraksiyonu (%EF) TBS’den otomatik olarak hesaplandı. Tiroid bezinin tutulum oranı (TUR) statik görüntüden hesaplandı.

**Bulgular:** IFN-β1a ile tedavi edilen ve edilmeyen tüm MS hastaları ile sağlıklı bireyler ötiroid idi. Anti-tiroid peroksidaz antikoru (anti-TPO), IFN-β1a ile tedavi edilen 15 MS hastasının 4’ünde (%26,6) tespit edildi. IFN-β1a ile tedavi edilen ve edilmeyen tüm MS hastaları ile sağlıklı kontroller arasında UR, EF ve TUR değerlerinde anlamlı fark yoktu (p>0,05). IFN-β1a ile tedavi edilen MS hastalarında TUR değeri, her iki kontrol grubundan düşük olmasına rağmen, fark istatistiksel olarak anlamlı değildi (p>0,05).

**Sonuç:** IFN-β1a tedavisinin, MS hastalarında KTBS ile tiroid ve tükrük bezi fonksiyonları üzerinde etkisi olmadığı gösterilmiştir. Tiroid ve tükrük bezi fonksiyonlarının tedavi edilmemiş MS hastalarında değişmeden kaldığı da bulunmuştur.

## INTRODUCTION

Introduction Interferon-beta (IFN-β) has been widely used to treat patients with relapsing-remitting (RR) multiple sclerosis (MS) (1,2). Since type I interferons (IFNs), mainly interferon-alpha (IFN-α) and IFN-β, modulate the immunoregulatory system, these cytokines may precipitate autoimmune disorders. IFN-β therapy has been associated with a relatively high risk of developing thyroid disease, as either organ dysfunction or autoimmunity (1,2,3,4,5,6,7). The incidence of thyroid dysfunction in patients treated with type I IFN is reported to be in the range of 1% to 35% in several studies (3,4). High incidence of thyroid dysfunction reaching up to 24% has also been reported in MS patients using IFN-β (2). 

Sjögren syndrome (SS) has been reported during treatment with IFN-α 2b for chronic hepatitis C and IFN-β therapy for MS (8,9). Recent advances have revealed a major role for activation of the type I interferon (IFN) pathway in the pathogenesis of the salivary gland hypofunction (10,11). It is known that alterations in salivary cytokines are seen in SS and that these abnormal salivary cytokine levels may contribute to the progessive destruction of salivary gland tissue in SS (11). 

Quantitative salivary gland scintigraphy (QSGS) using Tc99m-pertechnetate has been reported to be a particularly valuable tool for visual and quantitative assessment of gland function thus allowing assessment of the accumulation and the secretion of saliva (12,13,14). Salivary gland dysfunction due to various pathologies can be evaluated by this method. The main advantage of QSGS is the simultaneous evaluation of all major salivary glands and thyroid gland after a single intravenous injection, since the thyroid gland can easily be included in the imaging field of the SGS (14). 

However, to the best of our knowledge, no report has discussed the scintigraphically determined function of thyroid and salivary glands in MS patients treated with IFN-β. The aim of the current study was to assess the effect of IFN-β1a therapy on thyroid and salivary gland functions in patients with RR MS using QSGS. 

## MATERIALS AND METHODS

**Study Population**

A prospective, consecutive clinical trial was conducted. Fifteen MS patients (11 women, 4 men, mean age 35.1±8.3 years, age range 21-54 years) treated with IFN-β1a attending the outpatient clinic of the Department of Neurology were included in this study. All patients were affected by clinically definite MS and classified in the relapsing-remitting (RR) category according to McDonald’s revised criteria (15). All patients were clinically stable and off corticosteroid treatment for at least 6 months prior to the study. All patients were under IFN-β1a treatment for a mean of 3.50±2.28 years (range: 1-7 year with a minimum duration of 1 year). Of the 15 patients, 7 were getting a dose of 30 mcg (6 MIU) every week and 8 were getting a dose 44 mcg (12 MIU) every second day. 

Two control groups consisting of untreated RR MS patients and normal healthy individuals were included in the study. The untreated MS group who refused to start IFN-β treatment consisted of 15 patients (12 women, 3 men, mean age 33.2±7.0 years, age range 22-45 years) with demographic characteristics similar to the IFN-β1a-treated patients. The normal control group consisted of 20 euthyroid healthy individuals (15 women, 5 men, mean age 32.2±8.1 years, age range 22-48 years) with normal thyroid function tests and with no evidence of salivary gland dysfunction. 

All MS patients, treated and untreated with IFN-β1a, had no history of thyroid dysfunction prior to this study, and none had goiter on physical examination. This research was conducted by medically qualified personel in strict accordance with the guidelines of the Başkent University Medical Faculty Institutional Review Board, regarding the Tenets of the Declaration of Helsinki. All patients and controls gave informed consent as approved by our institutional review board. 

**Salivary Gland Scintigraphy**

After an overnight fast, dynamic SGS was performed with a large field of view single-headed gamma camera (ADAC, Argus, Philips) fitted with a low-energy, high-sensitivity, parallel-hole collimator with 140-keV photopeak for Tc-99m. The patients lied supine under the camera. The gamma-camera was positioned to include the head and neck region, so that the four major salivary glands and the thyroid gland was within the imaging field of view. Immediately after intravenous administration of 150 MBq Tc-99m pertechnetate, anterior sequential images of 1 min duration were acquired for up to 25 min. Dynamic images were digitally recorded in a 64x64 matrix with a 1.46 zoom. Salivary gland secretion was stimulated with 3 ml oral lemon juice (100% concentrated juice) instilled with a syringe at 15 min (13). At the end of the dynamic imaging, a static image of the same area was taken in the same position with 300.000 counts in 256x256 matrix and with a zoom factor of x 1.46. 

**Imaging Quantitative Analysis**

**Figure 1:**
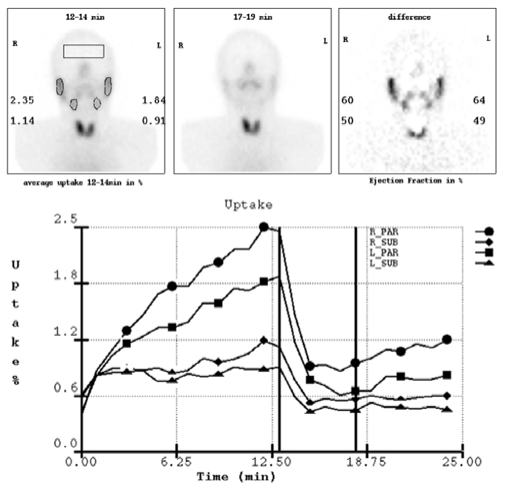
Quantitative salivary gland scintigram in a healthy individual. Regions of interest used for quantification are depicted on the left scintigram. Numbers on the left image represent uptake of 99mTc-pertechnetate in percentage of the activity (UR%) before applying lemon juice at 12-14 min postinjection. Numbers on the right figure represent the excretion fraction (EF%). Time-activity curves of all four salivary glands are demonstrated in the lower row.

All image acquisition and processings were performed by the same experienced specialist in nuclear medicine to ensure a meticulous and consistent technique. On all summation images of dynamic SGS, regions of interest (ROIs) used for quantification included one rectangular background ROI located over the brain and four irregular ROIs positioned over both parotid and submandibular glands (Figure 1). By computer assistance, time-activity curves (TAC) for the parotid and submandibular glands were generated. On the basis of these ROI counts on the TAC, the computer software calculated the following functional parameters for each salivary gland, as described previously (13): (1) uptake ratio (UR%): A measure of parenchymal function, that is the ratio of glandular Tc-99m pertechnetate activity at 12-14 minute post injection; (2) excretion fraction (EF%): A measure of secretory function, this value was calculated as the percentage of reduction in concentration after lemon juice stimulation. 

To evaluate thyroid uptake, we calculated the thyroid uptake ratio (TUR) as the thyroid-to-background ratio on the anterior static image. We drew the ROI for the thyroid manually by contouring both thyroid lobes. Background ROI for thyroid gland were placed just lateral to the thyroid ROI, as previously reported (14). 

**Labaratory Examinations**

Serum thyroid-stimulating hormone (TSH), free thyroxine (fT4), free triiodothyronine (fT3), anti-thyroid peroxidase antibody (anti-TPO), and anti-thyroglobulin antibody (anti-TG) were measured by chemilumminescent enzyme immunoassay system (Immulite 2000 BioDPC, Los Angeles, Calif., USA). 

**Statistical Analysis**

All thyroid and glandular parameters are expressed as mean value ± SD. Student t test and Mann-Whitney U test were used to analyze all mean values and to compare the three groups: healthy control individuals, untreated MS controls, and MS patients treated with IFN-β1a. Pearson test was used for correlation. All statistical analyses were performed using the statistical package for the social sciences software (SPSS, version 15.0; SPSS Inc., Chicago, IL, USA) for Windows, and a P value of less than 0.05 was considered statistically significant. 

## RESULTS

All patients with MS who are with or without IFN-β1a therapy, and healthy individuals were euthyroid. In contrast to control patients, anti-TPO was detected in 4 out of 15 (26.6%) patients (3 women, 1 man) treated with IFN-β1a. On visual evaluation of thyroid scintigraphy, homogeneous uptake within each thyroid lobe (no cold or hot regions) was observed in all cases. 

Comparison of scintigraphic parameters among the three groups are summarized in Table 1. No significant differences in the UR and EF values of bilateral parotid and submandibular glands, and TUR value of thyroid gland were found among MS patients with and without IFN-β1a therapy and healthy controls (p>0.05 for each). In addition, bilateral comparison of right -and left- sided glands in the same group revealed no statistically significant difference between right and left parotid and submandibular glands (p>0.05). Although the TUR values in MS patients treated with IFN-β1a were less than those of the both control group in this study, the difference was not statistically significant (p>0.05). We found no significant correlations between TUR and any of the salivary gland parameters.

## DISCUSSION

Interferon induced thyroiditis can manifest as clinical autoimmune thyroiditis, presenting with symptoms of classical Hashimoto’s thyroiditis or Graves’ disease, or as non-autoimmune thyroiditis (4). Non-autoimmune thyroiditis can manifest as destructive thyroiditis, with early thyrotoxicosis and later hypothyroidism, or as non-autoimmune hypothyroidism. It is believed that IFN induces thyroiditis by both immune stimulatory effects and by direct effects on the thyroid. 

The most common form of autoimmune type IFN-induced thyroiditis is the presence of thyroid antibodies without thyroid dysfunction. The titers of thyroid autoantibodies has been reported to increase in 40% of hepatitis C patients treated with IFN-α (3,16). In particular, a high incidence of anti-TPO without evident alteration of thyroid function has been observed in MS patients (5). We found that 26.6% of MS patients treated with IFN-β1a without preexisting autoimmunity had anti-TPO, indicating that thyroid autoimmunity occurs more frequently in MS patients treated with IFN-β1a than untreated MS patients and healthy controls. The prevalence rate in our study was consistent with those of previous studies (16,17). 

The measurement of thyroid Tc-99m pertecnetate uptake coupled with scintigraphic images of the gland provides valuable diagnostic information in patients with thyroid disease (18). Although thyroid scintigraphy has been widely used in MS patients with tyhroid disease, thyroid uptake analyses was not performed. In this study, we calculated TUR as a quantitative parameter of the thyroid gland function, as previously described (14). Although the TUR values in MS patients treated with IFN-β1a were less than those of the both control group in this study, the difference was not statistically significant. Previous reports demonstrated that IFN-β1a is less immunogenic than IFN-β1b, probably due to its greater structural resemblance to the natural human protein (19). A limitation of our study was the absence of TUR analyses before IFN-β1a therapy. Further studies with larger patient groups may be needed that compare scintigraphic parameters before and after IFN-β1a therapy. 

Interferons were also found to play a role in primary Sjögren’s syndrome, polymyositis, some forms of rheumatoid arthritis, and systemic sclerosis. Connective tissue diseases as seronegative polyarthritis and rheumatoid arthritis was observed even during early phase of treatment with IFN-β1a and IFN-β1b (20,21). The association between Sjögren’s syndrome and IFN-β has been reported (9), but the mechanism between IFN-β treatment and development of SS is unknown. A previous study has shown that type I IFNs and IL-6 can directly influence salivary gland function (10). In addition, some clinical trials with weekly intramuscular injections of α-IFN demonstrated improved salivary flow rates in SS patients (22). 

There is no study in the literature that evaluated the effect of IFN-β on the salivary gland in patients with MS. In the current study, the effect of IFN-β1a therapy on salivary gland function was assessed using QSGS. Quantitative SGS has gained widespread acceptance in the evaluation of a variety of salivary gland disorders in the literature (12,13,14). On the basis of time-activity curves generated from a dynamic study, a variety of different quantitative parameters have been proposed as a sensitive measure of main salivary gland function. In our study, two parameters was used for assessing parenchymal function and saliva excretion, as published previously (13). We calculated UR and EF as parameters for quantifying accumulatory and secretory salivary gland functions. In our study, no significant differences in the UR and EF values in both parotid and submandibular glands in patients with IFN-β1a therapy was found compared to untreated patients and healthy controls. We were unable to compare our results with those of other scintigraphic studies as, to our knowledge, this study is the first one related to this topic. 

A review of the literature reveals that there is only one study on the scintigraphic evaluation of salivary gland function in MS patients. Seze et al reported the prevelance of primary SS in primary progressive (PP) MS population as 16.7%, which is clearly higher than the usually accepted prevelence in the general population (23). In that study, the SGS, which is mainly based on visual analysis, was considered positive in 17 (28.3%) out of 60 patients. In 6 out of 10 (60%) PPMS patients with SS and 11 out of 50 (22%) patients PPMS without SS, SGS findings were suggestive of salivary gland dysfunction. However, the status of IFN therapy has not been mentioned in those patients. In relation to salivary gland function, we found no differences between MS patients without IFN-β1a therapy and healthy controls in our study. Different salivary gland findings between these two studies might be related to differences in study population and methods used in evaluating SGS (24). 

In conclusion, IFN-β1a therapy was demonstrated to have no effect on thyroid and salivary gland functions using QSGS in patients with MS. Thyroid and salivary gland functions was also found to remain unchanged in untreated MS patients. Future studies enrolling large numbers of patients will be required to confirm these findings. However, observation derived from the current study could be interesting for future trials aimed at detecting the effect of IFN-β therapy on thyroid or salivary gland function and encourages a multicentre prospective longitudinal study for addressing this question.

## Figures and Tables

**Table 1:**
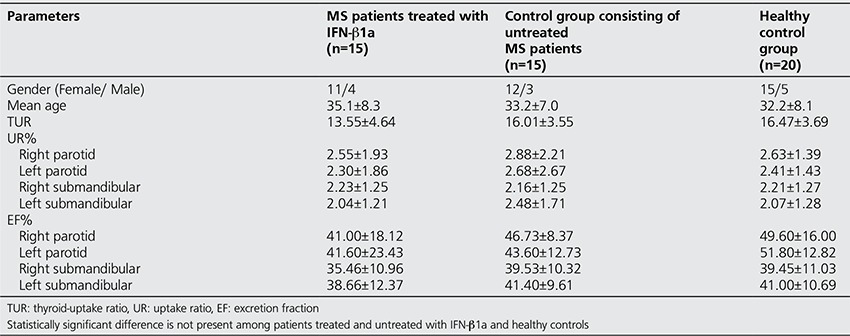
Comparison of quantitative scintigraphic parameters of salivary glands in MS patients treated with IFN-β1a, untreated MS patients and healthy controls
